# Women's Early Experiences with Online Stranger Sexual Harassment

**DOI:** 10.1177/10778012261418690

**Published:** 2026-02-03

**Authors:** Alisha C. Salerno-Ferraro, Caroline Erentzen, Regina A. Schuller

**Affiliations:** 1 Department of Psychology, 7991 York University, Toronto, Ontario, Canada; 2 Department of Psychology, 7984 Toronto Metropolitan University, Toronto, Ontario, Canada

**Keywords:** TFSV, sexual harassment, online harassment, gender, age

## Abstract

This research explored women's early experiences with male-perpetrated online stranger sexual harassment, including the age at which it occurred and its nature and impact. Most participants reported early experiences with unwanted sexual communication, most often beginning in their adolescence. Perpetrators were typically older males seeking a younger, pubescent girl to whom he would livestream masturbation, send dickpics or pornography, or offer money for sex. These communications elicited fear, disgust, anger, confusion around sexual development, and distrust of men. These experiences are prevalent and have enduring impact, reinforcing the need for policy, education, and clinical interventions.

## Women's Early Experiences of Online Stranger Sexual Harassment

Stranger sexual harassment has been studied for several decades as a phenomenon that occurs at street level, such as catcalling, following, sexual comments, remarks, flashing, groping and other unsolicited sexual communications (Fairchild & Rudman, 2008; Macmillan et al., 2000). The behavior is prevalent, with a large number of women reporting experiences with such behavior (e.g., Fairchild, 2023). The regular objectification and sexualizing of unknown women in public spaces have been described as a way for men to perform masculinity, reinforce gender power structures, and intrude into women's personal spaces (Baptist & Coburn, 2019; Vera-Gray, 2016). It is almost always interpreted negatively by the recipient, and regular exposure to it has been associated with self-objectification, body surveillance, shame, anxiety, fear in public spaces, fear of sexual assault and rape, poor mental health, PTSD, and suicidal thoughts and ideation (Carretta & Szymanski, 2020; Fairchild, 2010; Fairchild & Rudman, 2008; Frederickson & Roberts, 1997; Macmillan et al., 2000).

As a broad conceptual definition, sexual harassment involves unwelcome sexual behavior that interferes with a target's freedom or intended actions, which may include verbal statements, non-verbal behavior, gestures, comments, and requests (e.g., Henry & Powell, 2018). As the world has become more digital and connected online, stranger sexual harassment has evolved to find new ways to intrude sexually into women's personal spaces. Henry and Powell (2018) coined the term Technology-Facilitated Sexual Violence (TFSV) to refer to stranger sexual harassment that occurs in online spaces, including explicit text/direct messages, unsolicited graphic images, nude self-photos (cyberflashing), requests for sex, derogatory comments, and offers to exchange sex for money. The availability of internet access and the explosion of social media platforms and users have created unprecedented opportunities for strangers to access victims across geographic boundaries (e.g., Henry & Powell, 2018; Rentschler, 2014; Snaychuk & O’Neill, 2020). The actions are far from harmless, resulting in public humiliation, embarrassment, and hopelessness (Nilsson et al., 2019). In many ways, online harassment mirrors what was seen in street-level harassment. Catcalling on the sidewalk has become explicit texting; male flashing has moved from trench coats to dick pics. The present research sought to identify when such victimization occurs online, where it occurs, the age at which girls and women start experiencing online harassment, and its impact.

Adult women experience online forms of sexual communication from strangers relatively frequently, with more than 80% of university-aged Canadian women having regular experiences (Snaychuk & O’Neill, 2020). Blais and colleagues (2025) report that roughly one-fifth of adults admitted to having committed at least one type of TFSV, including cyberstalking, sexual harassment, and non-consensual sharing of intimate images. Powell and Henry (2019) found that 60% of Australian adults had experienced some form of TFSV. Flynn and colleagues (2024) similarly note that at least half of Australian adults have experienced TFSV, with roughly 25% experiencing stranger-perpetrated online harassment.

Less research has focused on how often such messages may reach younger girls in adolescence or teenage years or the age at which such sexual communications begin. This is concerning, as most youth in the United States, Canada, and the European Union are online daily (Anderson, 2018; Eurostat, 2023; Smith & Anderson, 2018; Statistics Canada, 2023). Teenagers report very high use of social media and digital networking (Smith & Anderson, 2018), suggesting a very high risk of contact with strangers and potential predators. The internet provides greater opportunities for anonymity and is associated with greater disinhibition than in-person interactions (Buchanan & Mahoney, 2022), potentially increasing the likelihood of harassment and child exploitation (Salter & Sokolov, 2024). This has serious implications for young girls, who may be exposed to sexually explicit images and messages from men who otherwise might not have approached them in-person.

### Objectification Theory and Gender Norms

Fredrickson and Roberts (1997) provide a clear theoretical explanation for the ongoing stranger sexual harassment of women by men. Under this theory, women and girls exist in a cultural environment in which their bodies are regularly appraised, monitored, and used for the satisfaction and pleasure of men. Some have argued that objectification of women also functions to maintain and support patriarchal power structures (e.g., Fairchild, 2023), which centers women's value around male attention and renders women unsafe in open spaces. The ultimate goal of stranger harassment is to make women feel unsafe and uncomfortable (Fairchild, 2023). As this harassment has moved into online spaces, it is likely that girls will experience male harassment in these spaces as well. Traditional forms of street-level harassment have been described as a ‘rite of passage’ for young girls, a phenomenon which they will almost certainly have to endure as the unavoidable right of men and boys to harass them (Saunders et al., 2017). As Fredrickson and Roberts (1997) observe, “with the changes of puberty, a girl's new body in a sense becomes ‘public domain’ (p. 193).

Traditional forms of street-level harassment have been described as a ‘rite of passage’ for young girls, a phenomenon which they will almost certainly have to endure as the unavoidable right of men and boys to harass them (Saunders et al., 2017). As the prevalence of online forms of harassment have extended the reach of male harassment of women, it is critical to understand just how soon this new “rite” is beginning and to assess its emotional and behavioral consequences. Baptist and Coburn (2019) note that men's intrusion into women's spaces has been normalized as a performance of maleness, an action which men do to show masculinity to other men at the expense of women's dignity and safety. Further, Vera-Gray (2016) notes that the fear instilled in women and girls by this performance of gender maintains a patriarchal gender hierarchy and continued oppression.

There are some who excuse the behavior as merely an attempt at flirtation, flattery, or ‘boys being boys’, but this characterization trivializes the harm such actions cause on their victims. Girls and women are expected to “roll with it”, ignore it, and not recognize the actions as a valid form of harm. Vera-Gray (2016) argues that it is ultimately a way for boys and men to perform power and dominance, essentially training girls to remain passive objects for male entertainment, using the term “stranger intrusion” to highlight the intended disruptive effects.

Most research on stranger sexual harassment contemplates that it is committed by a person unknown to the victim, with whom there is no prior social relationship, no professional or personal connection. These ‘strangers’ are encountered in public spaces (in-person or online), typically without warning, which may exacerbate the startling and shocking nature of the experience (Fairchild & Rudman, 2008; Henry & Powell, 2018). Such intrusions, note Baptist and Coburn (2019), never work to enhance the life of the victim, regardless of the supposed intention of the sender. These actions objectify, disempower, sexualize, and reduce women to bodies whose purpose is male satisfaction (Fredrickson & Roberts, 1997). These men are not seeking to start a conversation or see the woman as a human being with thoughts and knowledge.

In a recent literature review, Edwards and colleagues (2024) explored research on young people's (aged 10–25) attitudes toward violence against women. Attitudes toward sexual violence against women were related to beliefs about male physical strength, biological needs for sex, and heteronormative gender roles. Although most research showed that young people opposed violence and harassment of women, there are many situations in which such actions are normalized and justified, such as when it can be constructed as flirting or an attempt at romance. These circumstances may create confusion around sexual communications, particularly for inexperienced adolescents, although there is limited research exploring this question.

### Prevalence of Harassment and TFSV of Teenaged/Adolescent Girls

Previously, there was a conception of deviancy around adolescent sexting, interpreting it as a risky behavior analogous to drug use or unprotected sex (Mishna et al., 2021). However, non-consensual sexting is becoming recognized as a form of harassment, and an increasingly common one. A study of Canadian teens in 2019 revealed that 25% of boys and girls aged 12–17 had experienced online sexual harassment in the past year (Statistics Canada, 2023), although it is unclear what percentage of these incidents involved strangers versus peers. An international study of 14,000 girls in 22 countries found that 58% of girls and women aged 15–25 had been harassed or abused online (Plan International, 2023). This study also found that nearly one-quarter (22%) reported that they or a friend feared for their personal safety online, and one-fifth of girls have left social media or significantly reduced their use due to sexual harassment. A recent study found that 10% of girls aged 13–24 have unwanted sexual experiences online almost daily, with 40% at least monthly (Plan International, 2024).

Early studies on adolescent sexual harassment showed that this behavior has been prevalent for many years and is likely increasing in frequency. Ybarra and colleagues (2007) found that nearly 34% of teens aged 10–15 had been the victim of internet harassment, with 13% receiving sexual solicitation. Jones and colleagues (2012) found that 9% of teens aged 12–17 had received sexual solicitation online, 23% had been exposed to pornography against their will, and 11% had received harassment online. Mitchell et al. (2014) reviewed data from nearly 6,000 American teens aged 13–18 about their experiences with sexual harassment in the past year as a function of gender and sexual identity. At least 23% of teens had experienced sexual harassment in the past year, with significantly higher rates for transgender teens (81%), lesbian/queer girls (72%), bisexual girls (66%), and gay/queer boys (66%). These experiences were not benign, resulting in psychological distress and interference with school and work.

Douglass et al. (2018) surveyed 1272 Australian participants aged 15–29 about their experiences with stranger sexual harassment online, dating apps, smartphones, and social media. Eighty percent of women in the study reported experiencing sexual harassment in the past year, with 40% experiencing it on social media and 31% on their phone. Female participants were more likely to receive online harassment if they were under the age of 20. Englander (2012) found that more than one-quarter of late adolescent boys and girls had sent nude pictures consensually in relationships, but the girls were significantly more likely to report that they felt pressured or coerced into doing so. This resulted in a negative emotional impact and was more common among those who had experienced prior victimization. Similarly, Ybarra and Mitchell (2014) found that 7% of US teens aged 13–18 had shared a nude image of themselves online, with girls more likely to do this than boys. Girls were more likely to have engaged with someone older than themselves, and the sharing of such images was associated with lower self-esteem and other risky behaviors, such as having multiple sexual partners, substance use.

Mishna et al. (2021) assessed sexting practices and attitudes among 12–19-year-olds, in small focus groups. Most girls had received unwanted sexts, dick pics, or requests for sex. Most girls felt disgusted, nervous, scared and uncomfortable by the messages. Many reported difficulties expressing a lack of consent, feeling they had to explain their reasoning, negotiate their refusal, or make excuses. Many reported that boys would not accept no for an answer and continued to push them, blaming and emotionally manipulating them, becoming hostile, and blocking them if they declined to send a photo.

Ringrose et al. (2021, 2022) conducted interviews with 11–19-year-olds in England and Canada. Three-quarters of teen girls had received unwanted sexual solicitation, dick pics, and requests for nude photos. These were often from adult men they did not know in youth-oriented online spaces when they were 10–12 years of age. These men were appearing live and attempting to interact with the children; in other cases, girls’ snapchat conversations were interrupted by dick pics from strange men. All participants experienced this negatively, feeling disgust, fear, and embarrassment. This was often their first experience seeing anything sexual and it was interpreted as intrusive and violating. Some of the perpetrators were friends or acquaintances, which violated any relationship or friendship they might have had. Ricciardelli and Adorjan (2019) studied sexting and non-consensual dick pics in a sample of 13–19-year-olds in Canada. They found that the practice was so common among teenage boys that the girls had compiled enough photos to make a scrapbook to be shared at the end of their Grade 12 year.

### Harms of Technology-Facilitated Sexual Violence

Traditional conceptions of sexual and gendered violence have focused on physical acts of aggression, “leading to a trivialization of the kinds of harms effected through digitized communications networks” (Bailey & Burkell, 2021, p. 532). That is, there has been a perception that the digital nature of the harassment is not harmful, as one may simply ignore, block, delete, or not look at one's phone. In contrast, Kelly (1988) notes that gendered violence can take many forms and domains; women are exposed to many forms of violence throughout their lives (in private, public, professionally), which continue to reinforce their subordination by men. TFSV has been placed on this continuum of sexual violence (Harris & Vitis, 2020; Waling & Pym, 2019), acknowledging that unwanted sexual communication forces women into heteronormative intimacy with strange men and reinforces power and objectification.

Moreover, accumulating research has documented the significant and negative consequences of TFSV on women. This includes negative mental health consequences including distress, depression, alcohol use, PTSD symptoms, suicide attempts/ideation, and negative social and work consequences (Bates, 2017; Henry & Powell, 2016). After the initial TFSV event, victims may experience secondary harms such as gossiping, rumors, and stalking (Bates, 2017). The online nature of this harassment can make some forms of TFSV relatively permanent, especially where it takes the form of image-based abuse or so-called “revenge porn” (Dodge, 2016). Snaychuk and O’Neill (2020) found that women who had experienced TFSV reported poorer self-esteem, less sense of control and more depressive symptoms than those who had not had a TFSV experience. Champion et al. (2021) determined that TFSV increased the risk of suicidal ideation and affect through the feelings of bullying. Champion et al. (2022) further found that women's experiences with TFSV were associated with poor mental health outcomes, including stress, anxiety, depression, loss of trust, alcohol use, and impairment at work or school.

The degree of psychological harm varied by the nature and prevalence of the abuse, with the most negative outcomes resulting from image-based abuse. Klettke et al. (2019) studied young adults’ (18–21) experiences with sexting, finding that receiving unsolicited and unwanted sexts (sexual texts) or being coerced into engaging in sexting was associated with depression, anxiety, lower self-esteem, stress, and overall poorer mental health. Less is known about the mental health consequences for young girls and teens, for whom stranger sexual harassment may be their first experience with sexual images. Oliver and colleagues (2024) connected online sexual harassment of women with objectification theory effects, observing that the increase in disordered eating and alcohol misuse caused by TFSV was mediated by increased self objectification caused by the TFSV. What effects might this have for their developing sense of sexuality, trust in men, or sense of personal privacy and safety online?

### Present Research

The present research expands on this emerging literature, assessing early experiences with stranger sexual harassment online. Informed by objectification theory (Fredrickson & Roberts, 1997), the present research investigated online stranger sexual harassment, assessing the general age at which it first began, the nature of the communication, and its effect on the user. Using a mixed-methods approach, we assessed women's self-reported early experiences with TFSV, including the location and frequency of early harassment as well as a thematic analysis of their descriptions of their earliest recollected encounter. Although we maintained an exploratory lens, we anticipated that online forms of sexual harassment would mirror those seen at street level, beginning around the age of puberty, being an experience common to many women, causing negative emotional responses rather than positive ones. We further anticipated that victim narratives would reflect patriarchal power dynamics in which men felt demonstrated entitlement to girls’ bodies, sexualizing them at an early age, and reinforcing power lines along which girls must learn their position as objects of male sexual desire.

## Method

### Participants

Four hundred women were recruited from York University in exchange for partial course credit. Participants were excluded if they identified as transgender (*n* = 1) or non-binary (*n* = 2), resulting in a final sample of 397 participants. The ages ranged from 17–48 years (*M* = 19.78, *SD* = 3.21). As seen in Table 1 there was racial and religious diversity in the sample. The majority of participants identified as having a heterosexual orientation (*n* = 336, 84.63%), with 9.32% (*n* = 37) identifying as bisexual and a smaller number identifying as lesbian (*n* = 1, 0.25), another sexual orientation (*n* = 8, 2.02%), or preferring not to disclose (*n* = 12, 3.02%). Institutional ethics approval was obtained from the York University Research Ethics Board (e2019-373). Informed consent was obtained from all participants.

**Table 1.** Demographic Characteristics of Participants.

		N	%
Ethnicity			
	Black	61	15.37
	East Asian	54	13.60
	Hispanic	7	1.76
	Middle Eastern	44	11.08
	South Asian	115	28.97
	White	68	17.13
	Multiracial	34	8.56
	Another Race	13	3.27
	Not disclosed	1	0.25
Religion			
	Atheist/Agnostic	69	17.38
	Buddhist	13	3.27
	Christian	118	29.72
	Hindu	36	9.07
	Jewish	3	0.76
	Muslim	107	26.95
	Sikh	29	7.30
	Multifaith	3	0.76
	Another faith	19	4.79
	Not disclosed	0	0.00

### Materials and Procedure

Participants were invited to voluntarily take part in this study for partial course credit. Those who signed up were told that the study would explore their experiences with stranger sexual communications online. This was part of a larger study on stranger sexual harassment that had occurred in the past two years, with the final portion of the study assessing early experiences. After completing their adult victimization experiences, participants were presented with two questions which asked, “How old were you when you first experienced unwanted sexual attention from a male stranger online? Can you tell us a bit about what happened?” Participants could then provide a description of such an experience in their own words. These responses were reviewed and coded for any mention of their age at the time and the location/platform on which it occurred. Descriptions of the harassment were provided by 244 participants (61.46%). Another 101 participants provided only the age of their earliest recalled experience with no further description (25.44%), 5 participants reported that they were not comfortable disclosing or could not provide details (1.26%), 21 participants did not answer the question at all (5.29%). Only 22 participants (6.55%) said that it had not happened to them.

The data correspond to a two-year period between November 2018 to November 2020. The term ‘unwanted sexual attention’ was selected for this research question rather than ‘sexual harassment’, as the latter term is a legal concept under both criminal law (e.g., s. 246 *Canadian Criminal Code*) and civil law governing workplace contexts (e.g., Title VII, Civil Rights Act, 1964). The term sexual harassment may have indicated to participants a higher threshold of harassing behavior, criminal experiences, or a workplace context, which was not the scope of this work. The term ‘unwanted’ was included to ensure we are not capturing consensual or reciprocal sexual communications, but to focus on unsolicited sexual attention from a stranger.

## Results

Three hundred and twenty-four participants provided a specific age at which they recall their earliest online stranger harassment. The average age at which online harassment began was 15.14 years (*SD* = 1.58, range 7–27). Three participants reported that they were 7–9 years of age (1.23%), 41 reported that they were between 10–12 years of age (10.33%), 80 were between 13–14 years (24.69%), 157 were aged 15–17 (48.46%), and 42 were 18 or older (10.58%) when the harassment began for them. Some participants also reported the location, app, or platform on which it occurred. Most participants (*n* = 117) did not specify the precise location/platform, but of those who did include location information, the most frequently reported application was Instagram (*n* = 46), followed by Facebook (*n* = 14), Omegle (*n* = 10), Snapchat (*n* = 8), Kik (*n* = 6), Discord (*n* = 2) or a dating app (*n* = 12).

### Thematic Analysis of Narratives

Open-ended responses were analyzed by the authors using thematic analysis (Braun & Clarke, 2006, 2019) to identify themes or patterns that repeatedly occurred. An inductive approach was used to analyze the responses. In line with Braun and Clarke's (2006) guidelines, the authors began by immersing themselves in the dataset, reviewing participants’ responses and familiarizing themselves with the data. The entire dataset was then systematically coded, identifying patterns and segments of data that appeared relevant and meaningful. The authors then met to compare their codes, discuss any discrepancies and reach consensus on the coding.

The authors collaboratively collated codes into potential themes by grouping codes that shared common features. These initial themes were broad and served as a starting point for further analysis. The potential themes were then reviewed and refined by the authors, checking if they worked in relation to the coded extracts and entire dataset. Finally, the authors worked together to develop clear definitions and names to capture the core meaning of each theme. Responses were also analyzed to extract straightforward data such as the age at which the harassment occurred and the location of the incident (e.g., social media website, application). This descriptive coding was conducted to categorize and summarize demographic and specific-event-related information accurately. This was done by two independent research assistants, using a codebook developed by the authors. A summary of these themes and examples of relevant statements are provided in Table 2. Note that some comments may discuss multiple themes and example statements may touch on more than one thematic idea. As outlined more fully below, three overarching themes were identified with several sub-themes in each. For most participants, these incidents occurred during adolescence. In keeping with the principles of thematic analyses (Braun & Clarke, 2006, 2019), we report themes descriptively along with illustrative quotes, rather than quantifying the frequency of particular experiences. This approach preserves the richness of participants’ narratives and highlights shared patterns of meaning without reducing them to counts.

**Table 2.** Themes and Illustrative Quotes from Open-Ended Descriptions of First Harassment Experience.

Theme	Illustrative quote
Older Men Seeking Young Girls	
Older men	I was 12 years old on facebook. This man messaged me saying how beautiful and pretty I was, but he seemed to be an older man and I was just 12.
Sexualizing puberty	When I was 6 years old, first time using social media. I remember he asked me for nude pictures of my breast. I was 14…he asked to take my virginity
Sugar daddies	…around the age of 13 years old. Around that age I made my Instagram account and I would get message requests from guys that were 3× my age asking for sexual photographs or asking for sexual things in exchange for money.
Entitlement and Intrusion	
Persistence and escalation	I was in grade 9 when a random stranger began messaging me inappropriate messages on Instagram. I blocked the user but they made new accounts and continued to message me. The person even began to message my friends and ask why I was not replying.
Unsolicited and intrusive	I was around 15 and guys would dm me on Instagram even though I have a private account and tell me im gorgeous and want “bob pics"
Personal threats	…maybe 10 or so, I received an Instagram DM from an old man asking my age and not to tell my parents about us messaging. …he said he knows my teachers…
Reactions to harassment	
I thought we were friends	I was 15 and just signed up for Facebook. These older men started talking to me and I would respond back in the sense of being friends, but they would take it the other way and sexualize everything. In the end, I blocked them and limited my posts and interaction with strangers on social media.
Seeking support from girls	…if a bunch of messages pile up, whenever my friends and I get together we go through them …I told my older sister and she told me if I ever encounter people like this I should just block them
Fear of Posting	…when I was 14…I used to post a lot more on Instagram and random males would comment and private message me and say inappropriate things. It was disgusting and because of that, I don't have any posts now and barely want to post (I'm 20 now and I still hesitate to post).

### Older Men Seeking Young Girls

Participants consistently described encounters with adult men who targeted underaged girls online, emphasizing a significant age disparity that created a clear power imbalance. These interactions often involved sexualization of participants’ bodies during puberty, unsolicited propositions such as “sugar daddy” offers, revealing a pattern of exploitation in digital spaces. Collectively, these sub-themes illustrate how age and perceived authority of the perpetrators, coupled with sexualized attention toward adolescent girls, created a persistent and invasive form of harassment. These findings highlight the heightened vulnerability of young girls online and the ways adult men exploit this vulnerability for sexualized or transactional purposes.

**Older men.** The older age of the perpetrators was a salient aspect, with many participants explicitly referring to them as middle-aged or characterizing them as a “grown man” or “old man”. Importantly, participants emphasized that these individuals were not just slightly older but significantly older than themselves. This was not a situation in which girls were targeted for harassment by teenage boys or peers, but by much older adult men who were seeking out underaged girls. One participant shared a poignant account of creating an Instagram account at 13 and receiving inappropriate messages from individuals three times their age. One participant recounted a case wherein “*I was 12…weird grown men like in their 30 s and 40 s would dm me asking for pictures or trying to flirt/sext*”. Another participant mentioned a time she “*was 13/14 years old. I was on Omegle and an older man (30/35) showed me his genitalia. I was scared. I didn’t even know what sex was*.”

Another participant shared a disturbing encounter that she experienced at the age of 10, on the now defunct virtual chatting room Omegle, which would randomly pair users in one-on-one video chat sessions where they could chat anonymously. As she described the encounter, “*When I was about 10 me and my friend went on Omegle and were met with a middle aged man with his penis fully erect on the screen. We were so in shock we just stared at the screen in disbelief. He then went on to rub himself asking us if we liked what we saw saying a few gross things before we quickly exited out of the app. It was traumatizing and we both cried afterwards, worried that our parents would find out (like it was OUR fault)*”. These encounters, marked by a significant age disparity, highlight a power dynamic that contributes to the vulnerability of young women navigating the digital landscape. The explicit nature of these encounters further intensifies the distress, creating an environment where young women, sometimes prepubescent girls, are confronted with inappropriate and potentially harmful content without their consent.

**Sexualizing puberty.** A few respondents discussed disturbing encounters where they were met with inappropriate comments that centered around sexual development and virginity. These comments were directed to girls when they were in their adolescent years and suggest that men were sexualizing their experiences with puberty in an overt manner. For example, “*I was around 15 when I first received a sexual comment about my body because I was getting boobs from puberty so this guy pointed out how my chest wasn't flat anymore and how he liked it*”. Another participant recounted the following: “*I was 14 years old. I received a DM from a guy at school saying he thought he was physically attractive and (I'm paraphrasing a bit) ultimately asked to take my virginity*”. Many of the comments pertained to their bodies as being not quite childlike, yet none showed appreciation for them as maturing into adulthood. The fascination and sexualization attached to their proximity to childhood and adolescence.

One participant described a disturbing experience she had on Instagram as a preteen, “*I think I was around 12 or 13 when I first got instagram, had a really small following and an un-private account. it was always old men and pedophiles that would message me or try and engage into conversation knowing I was a minor. One guy intensely described all the things he would do to my ‘child like’ body and that it ‘turned him on’. from them my account was private and has been all these years*”. One participant mentioned a time when she was 15 and her mother posted a picture of her on Facebook. An older man commented under it saying that she was “*all grown up now but it was definitely not in a friendly way. He commented under the photo many times*.” In one particularly disturbing account, a participant indicated that she was just 6 years old when a stranger asked her to send a picture of her breasts. The focus on girls’ secondary sexual characteristics reflects not only the sexual interest but also the social construction of girls as simultaneously childlike and sexualized. The sexualization of puberty underscores the broader cultural dynamics that normalize viewing girls’ bodies through a lens of male entitlement.

**Sugar daddies.** Another common theme emerging from the women's early experiences was that of encountering “sugar daddies”, or men offering financial support in exchange for sexual favors. These men engaged in deliberate and somewhat predatory efforts to find young girls online and to offer them money or gifts in exchange for sexual access. This included sending girls images of pornography, showing themselves masturbating, or asking the girls to perform sexual acts or send picture of themselves. Some participants explicitly described these men as sugar daddies, for example, “*I think I was about 15 years old when I associated myself with more than one social media app. The unwanted attention I got back then was a male stranger asking me to let him be my ‘sugar daddy’ or sending me pornographic images*”. Others described encounters where they were offered gifts in exchange for sexual attention. One participant described a man offering to pay her to be in a sexual relationship with him, “*the first time I started receiving messages was when I was 18. It was an Instagram direct message from a man who appeared to be quite old, and he wanted to send me money in exchange for me meeting him and doing things with him. It made me feel nervous and afraid, I blocked and reported the account, and deleted his message*”.

The participants’ expressions of shock and surprise in response to sugar daddy requests underscores a fundamental disconnect between their intentions in online spaces and the predatory nature of these propositions. What becomes apparent is that many participants were not actively selling or advertising any form of intimate services, yet they found themselves confronted with explicit proposals linking financial incentives to sexual favors. The surprise expressed by respondents highlights the unsettling realization that, for some strangers, the assumption prevails that sex is a transactional commodity available for purchase, irrespective of consent or intent. This aligns with broader patterns of hegemonic masculinity, where men's sexual entitlement intersects with economic and social power to exploit vulnerable populations.

### Entitlement, Intrusion, and Threats

A recurrent pattern across participants’ narratives was the sense of entitlement exhibited by perpetrators, who often ignored rejected and escalated harassment when refused. Sub-themes such as persistence and escalation, unsolicited and intrusive sexual messages, and explicit or implied personal threats coalesce to depict a pervasive disregard for young women's boundaries. These behaviors convey a broader message to victims that their autonomy and safety are not respected, illustrating that the emotional and psychological toll of navigating online spaces where harassment may be aggressive and continuous. The persistence and escalation of harassment, as well as the violation of boundaries through unsolicited sexual messages, reflects underlying norms of male sexual entitlement. These behaviors can be interpreted through the lens of hegemonic masculinity, where man assert power and dominance, often disregarding consent, to reinforce gendered hierarchies in both online and offline spaces.

**Persistence and escalation.** Some participants characterized their encounters as persistent, noting that even after rejecting or ignoring the perpetrator, he continued to harass her. For example, “*I believe I was 14. A person that I do not know kept sending me inappropriate pictures of him. Even though I blocked him, he kept sending messages and pictures from other fake accounts.”* Some participants indicated that the men would become angry or upset when their persistence was met with rejection, as the following participant recounts, “*…he would just make new accounts to send rude comments and would get angry when I would not answer”.* This sense of not taking no for an answer reflects an inherent understanding that stranger sexual harassment is built on a feeling of entitlement to girls’ attention and spaces.

Others noted that the men were not only persistent but would become angry or threatening upon refusal. “*If I refused to answer a question or the men didn't like my answer, they would become angry or insult me. It's interesting now when I think back that I would somewhat take it personally if I made these strangers angry or upset. I would sometimes lie in order to change their opinion from negative to positive. It can be really dangerous, especially for young, impressionable girls who are seeking approval.”* These persistent and sometimes aggressive communications communicate the message that it is not acceptable for a girl to reject a man's sexual interest and that their own personal boundaries are not respected.

**Unsolicited and intrusive.** Participants described instances where sexually inappropriate or explicit messages were received, unsolicited and without warning. For example, one woman recounted the following: “*I would start chatting with a stranger and they would ask “a/s/l”? (age/sex/location). I would tell them how old I was and regardless, they would eventually lead the conversation to asking me what my bra size was, what kind of underwear I was wearing, what did I look like etc.*” Another woman reported she received a proposition from a random man on Snapchat when she was 16 who “*said that the relationship could be purely physical, and he asked if I’d like to help him realize his fantasies*”. Some respondents described the messages they received as more explicit and descriptive, for example, “*the dude kept complimenting my appearance and told me in very descriptive language of what he wanted to do to me and also tried to act out the scene he had in his head through text messages*”. Many women characterized the messages they received as creepy or disgusting. For one participant, when she was 14, “…*I used to post a lot more on Instagram and random males would comment and private message me and say inappropriate things.*”.

One prominent theme that emerged from the data revolves around the receiving and solicitation of unsolicited nude photos and video content. Participants shared stories of being sent unsolicited nude pictures, as the following participant described, “*the first time I got sexually harassed I was 12. I was new to social media and was very excited when I got a message request from someone I did not know. To my dismay, I was met with an unsolicited nude picture of a man. It was very disturbing*.” Many of the nude photos that participants described receiving was specifically penis-related content, like unsolicited “dick pics”, and in one case, a man masturbating on video chat. As the participant explained, “*he messaged me a few times and I responded because I didn't want to seem rude, a couple of days later he sent me a picture of his dick and I blocked him*”. Similarly, some participants talked about their experiences of being solicited for photos rather than being sent them, for example, “*I was most likely around 14 years old when I was first asked to send a stranger nudes*”.

**Personal threats.** Some participants recounted experiences in which the sender made vague threats and implied that they knew the victim personally, exacerbating the fear and harm of these encounters. “*I was in 7th grade so around 12–13 and I got an older man who offered to give me gifts or money if I met him and had sex with him. I told him I was too young and he said he knows my teachers…I’ve had my socials on private since then*.” Another participant described a scenario in which she was 15 and received “*…a message on Facebook that was threatening. A man told me if I didn’t send him pornographic images he would leak my banking information - I immediately knew it was a scam but it still made me feel uneasy*.” Yet another participant described an experience when she was 14 and received “*multiple texts from older men. Once an older guy kept asking me for my pictures so I blocked him. Later I found out he was using girls pictures to blackmail them. It scared me a lot*.” These threats suggest to the girl that her identity has been compromised, that she does not have the option of refusing male sexual interest, and that she is never safe in online spaces.

### Participants’ Reactions to Harassment

Participants’ reactions to harassment reflected a combination of emotional distress, adaptive strategies, and reliance on social support. Common emotional responses included fear, shock, anger, disgust and shame, with some participants describing long-term effects such as altered patterns of online engagement. Coping strategies ranged from blocking or reporting perpetrators to seeking guidance. These reactions reveal the non-benign impact of online harassment of young girls’ well-being, illustrating both the immediate distress and enduring consequences of such experiences. Collectively, these responses underscore the importance of understanding v ictim experiences not just in terms of exposure to harassment but also in terms of the ongoing effects on emotional and social development.

**I thought we were friends.** Some participants described instances where they were engaging in seemingly friendly conversations with strangers they had met in chat rooms, or on social media, only to be confronted with an unexpected escalation into sexual dialogue. Participants recounted experiences of building what they believed to be genuine connections, sharing interests, and forming a virtual friendship when suddenly, the tone abruptly shifted toward explicit and sexual content. For example, “*I used to talk to a person in Yahoo chat and we used to talk normally. He was a doctor and was a married man, much older than me. We talked as friends for a couple of days, until one day he asked me something sexual. I don't remember what he asked. I cut off ties with him immediately*”. Another participant recounted an experience when she was “*around 16 years old. I was talking to a friend of a friend when he confessed his feelings for me. When I said I was not interested, he kept talking about sexual stuff he wanted to do. I was disgusted and ended up blocking him*.”

The narratives within this theme highlight a notable ambiguity surrounding the intentions of the senders, leaving the nature of the relationship unclear for the young women involved. Given that many of the women were young girls in these described encounters, it is possible that they did not fully grasp the subtle cues that signal disguised sexual intentions. In many instances, these young women, being children, may have interpreted seemingly friendly conversation as genuine connections unaware that they were veiled attempts at initiating romantic or sexual engagement. For example, one participant recounted an experience with an older male: “*I was about 14, and I was getting used to Instagram when a young-looking male texted me and I thought he wanted to be friends. He was actually 18 and he was nice at first but he randomly started sending inappropriate comments*”.

**Seeking social support from girls and women.** A number of victims mentioned that uncertain of how to react or proceed, they sought or received help from an older girl/woman in their life who had been through a similar experience. For example, one participant described an experience when she was 14 in which an older male was sending “*…inappropriate comments. I told my older sister and she told me if I ever encounter people like this, I should just block them*.” Another participant described a time when she was 12, and a middle-aged man sent her a penis picture to her messages. “*I cried and told my aunt to delete it.*” Others mentioned that they learned that this behavior is commonplace among their peers, that “*I heard that these things happen from my friends.*” Another participant stated that she had private accounts to avoid such contact, but a person she knew sent strange messages from a man who *“would send posts to many girls cause some of my friends received the same message as well*.” Another noted that “*In high school, some friends found it funny mainly because they would get similar messages. They’d urge me to block him*.” Another girl described a time she was 15 and approached by a male in Grade 12 (18 years old), who “*was known for asking younger girls to do things for him.”*

**Emotional responses.** Although participants were not explicitly prompted to report their reactions to the harassment, many voluntarily expressed how they felt after being harassed in an online space by a stranger. These different emotional responses are presented in Table 3 along with an illustrative quote. The most common emotional reactions spontaneously identified by participants include fear and shock, anger, annoyance, disgust, discomfort, and shame. No participant reported having a positive or favorable emotional reaction. Notably, respondents did not interpret these experiences as mild or innocuous, and some expressed feeling quite disturbed by the content they were sent. For example, one respondent reported: “*I had just turned 13 when a boy twice I my age sent me unsolicited dick pics and asked me for nudes. I was and still am to this day traumatized, and still, get traumatized every time I receive one*”. Some participants reported changing the way they used social media, for example, “*It scared me and made me uncomfortable how these men were on an app that was mostly used by younger age groups so I never posted a picture again and just strictly used Kik for messaging people I knew*”. Another participant recounted “*I was also really young and not expecting that, I decided to change my profile to private as I thought fewer strangers would message me then*”. Also of concern, these effects appeared to create long-term distrust of men and confusion around their safety in online or public spaces where they may encounter men. These responses not only shed light on the immediate negative impact of women's early experiences with online stranger harassment but also underscores the enduring, long-term consequences of these encounters.

**Table 3.** Common Emotional Responses to Harassment Described by Participants.

Emotion	Illustrative quote
Fear and Shock	[I was] around 7 or 8 years old, it was a guy 5–6 years older than me, he was trying to talk to me about sex, using a bunch of disgusting phrases. I ignored him, but I was extremely scared and ashamed.
Anger	14. I do not remember very clearly but I do know this was somebody messaging me from the country I was born in and certainly someone older who was very direct in terms of what he wanted, what he wanted to do with me. My reaction was of shock and anger and disgust. I told this person off and blocked them I believe.
Annoyed	I was around 16. A boy in my high school that I didn't really know at the time had added me on Snapchat and flattered me with many comments about how pretty I was and mentioned my body. It made me feel awkward and annoyed at the situation. I politely told him to stop and he did. We don't talk that much anymore, sometimes on occasion.
Disgust	16, this guy messaged me on Instagram and was saying stuff like we should hook up, lets meet up, and I was honestly disgusted
Discomfort	Probably at the age of 14, they would try and start a conversation or compliment me, but it made me uncomfortable as I didn't know them and they were much older.
Shame	The first time I had received an unsolicited picture from a man of his genitalia was when I was 12. It was on Kik. I immediately deleted the picture and blocked the man who sent it. I was so ashamed and afraid to tell anyone because I thought they would look at me differently and I didn't want my parents to monitor my social media. I thought it was my fault and I blamed myself for receiving the picture. I didn't tell anyone up until last year.
Distrust of Men	I was 12…imagine my young mind seeing a penis photo sent by a middle aged man through Facebook messages…that particular facebook account remains untouched and I have been scared of middle-aged men for the longest time.

## Discussion

This research provides insight into online sexual harassment of young adolescent girls, including how early it begins, common themes observed in this form of harassment, and its negative effects. A prevalent theme involved older adult men seeking sexual interactions with young girls, very clearly children in youth-oriented spaces. This harassment intruded into their personal and seemingly private online spaces, forcing sexual images and ideas into their minds. It was uniformly received as unsettling, disgusting, and frightening and in many cases led to long-term behavioral monitoring and consequences, such as changing the way they use social media. This could certainly not be characterized as minimal harm simply because it occurred online, nor should it be dismissed and excused as boys being boys. The degree of intrusion and harm this harassment caused requires serious response and consideration.

Hayes and Dragiewicz (2018) found that men who send unsolicited dick pics are often doing so out of a sense of sexual entitlement to the victim's attention and sexuality, as a form of sexual violence or aggravation, or a way to repair damaged masculinity. This aligns with work by Oswald et al. (2020), who found that the most common motivation stated by those who send “dick pics” is a hope for reciprocation or sexual contact, with a minority sending the images out of hostility and misogyny, gratification that a woman was forced to see his penis against her will, or a desire to make the woman angry, shamed, devalued, or fearful. Karasavva et al. (2022) recently replicated these findings, also determining that although the transactional mindset was the most common reason given for cyberflashing, more than 40% of senders anticipated a negative response from the recipient – that they expected to be ignored, mocked, or to elicit anger. Moreover, cyberflashing due to power/control motives was associated with higher psychopathy, personal gratification motives were associated with narcissism, and sadism was associated with the hope of receiving an image back but not a partner.

### Implications

There are larger implications of this early and regular harassment of girls, who may learn that certain online spaces are not acceptable or safe and who may learn to fear all men as potential predators. Jiwani et al. (2010) note that structures and systems of violence can systematically exclude communities from certain spaces. Professional expressions may result in male objectification; online gaming may result in harassment and degrading language. Eventually, girls may self-select out of such spaces, losing enrichment, self-expansion, and recreation in a way boys and men do not, “thereby also restricting access to the social, cultural, and economic benefits of that participation” (Bailey & Burkell, 2021, p. 534).

Boys and men may also be concerned about the digital footprint they are leaving behind, particularly where they can send intimate messages instantaneously and permanently. In some cases, men may unknowingly engage in sexual communication with a child or young teenager, potentially distributing pornographic images and child sex abuse materials. There are serious legal consequences for such actions, suggesting that a much stronger degree of personal responsibility and diligence is required as well as enforcement and oversight by third parties.

#### Legal Reform and Policy

In 2018, the United Nations called for laws and legislation to deal with online sexual harassment targeted at women and girls, noting this to be a new way in which their human rights are being violated (United Nations, 2018). There does appear to be a growing movement toward legislating these actions. For example, with the newly enacted *Online Safely Act* in England, a man was recently sentenced to a year in prison for cyberflashing, sending unsolicited photos of his genitals to a 15-year-old girl and an adult woman (Javaid, March 20, 2024). California allows victims of such cyberflashing to seek financial compensation of up to $30,000 from the sender in civil court. In 2019, Texas passed legislation to prohibit the sending of unsolicited nude photos without consent of the sender, making such an action a misdemeanor (Texas Penal Code 21.08). Canada has had, since 2014, the *Protecting Canadians from Online Crime Act*, which provides an alternative legal route for dealing with youth under the age of 18 who disseminate pictures (otherwise it would be child pornography).

Baptist and Coburn (2019) highlight the need for recourse for victims of stranger harassment, noting that few remedies exist. Because this is usually committed by anonymous strangers, it is hard to identify or prosecute these actions. Many responses are ex post facto, providing options or consequences only after the harassment has occurred rather than developing proactive strategies that may deal with larger structural factors (Bailey & Burkell, 2021). There is a lack of attention and accountability toward corporations and social media platforms who facilitate and overlook the thousands of actions of harassment and abuse that occur daily, prioritizing capitalist gain over safety (e.g., Salter & Sokolov, 2024). There is a clear need for legal avenues to pursue action against social media platforms, with civil and potentially criminal liability that may affect profit margins and thus lead to better enforcement.

#### Non-Legal Responses

Other effective strategies might involve naming and shaming perpetrators, exposing their names and actions, recording and reposting their messages, and sending them back to their parents, employers, or partners (Fileborn, 2014; Harris & Vitis, 2020). Hashtag activism such as #Metoo (Clark, 2016; Powell & Henry, 2018) can be effective at making larger social changes and educating men on the harmful impact of this unwanted and harassing behavior. It should be noted that victims who speak out put themselves in potential risk of retaliation or danger, despite any empowerment they may receive (Harris & Vitis, 2020). Jane (2016) notes also that this so-called “digitalantism” can lead to scapegoating of individuals without targeting larger structures of power and oppression and larger feminist goals. Fileborn and Vera-Gray (2017) note that there is some justice for survivors through online shaming and webpages, but it can come at a cost of revictimization and silencing. Others note that seeing women sharing their experiences on twitter can be a form of solidarity (Fileborn, 2021). Allyship and confronting sexual harassment may be another extension of this general approach.

Other efforts might incorporate more women into the platform development and oversight. Women and racial minorities are underrepresented in Artificial Intelligence, digital platforms, and social media, resulting in a disconnect between design and user experience and a reinforcement of existing cultural biases and power structures (Buolamwini, 2019; Ekman, 2019). We need not just a shift in diversifying platform creators and tech industry, but also a movement toward societal intolerance of gendered harassment and violence. Diversifying tech, however, is not sufficient in itself if the social power imbalances and problematic gender norms are not also addressed (Hampton, 2021). Zuboff (2019) notes that state-based legislation that regulates the tech industry against ongoing negligence may provide some opportunity. In other words, where corporate profits may be affected by lawsuits and fines, changes may be quicker to come than simply waiting for male dominated fields to care about harm to girls and women.

Mishna et al. (2021) found that many teens had exposure to unsolicited sexts and dick pics and struggled with issues of consent and boundaries. They noted a common theme that adults were not involved in their online world or experiences, suggesting that a greater role of parental oversight would be appropriate to help navigate these regular non-consensual sexting experiences. As a key recommendation, they note that young children and teens should be kept off social media apps as much as possible. Parents should not rely on app protections, parental controls, or the good judgment of kids or the strangers who may find them. This is particularly a concern as many social media apps have geolocation functions and many girls and women have their phones near them at all times (Porter et al., 2025). Although apps may expressly prohibit sexually harassing or explicit communications in their user guidelines, they are often reluctant to take responsibility for user conduct (e.g., Albury et al., 2017; van Royen et al., 2015). Responses may include clearer and stricter standards of what constitutes harassment as well as clear and visible procedures for reporting (Porter et al., 2025). We suspect that social media platforms might find their way to effectively address harassment if they were subject to financial liability for the psychological harm their platforms enabled under their watch.

It is imperative that schools develop policies to manage social media and technology use, particularly as it creates a risk of harm and sexual violence. Elementary, middle, and secondary schools should create better reporting mechanisms, provide support for victims, and mental health resources and screening of victimization. Students who harass their classmates should face consequences, with clear reporting mechanisms for victims. Clinicians and schools need to ask about these experiences, assume that they are happening, and that they cause harm.

### Limitations and Future Directions

One potential concern with the present research involves the use of university students as participants. This population was chosen, however, as young adult women experience the highest rates of TFSV (Henry & Powell, 2018). Given that the study was investigating early experiences with digital harassment, we did need women who were young enough to have had these experiences when the internet was a fairly ubiquitous presence in their lives, thus excluding older women samples. We note, however, that the prevalence of online harassment reported by young women in university is consistent with those not in university across a number of samples (e.g., Reed et al., 2020). Moreover, given the widespread nature of cellphone usage, it is unlikely that a university-based sample would differ significantly from a non-university sample with respect to their experiences in adolescence, although future research may wish to investigate early experiences with a broader sample.

In addition, we note that these accounts relied on autobiographical memory, which may be subject to some distortion or inaccuracy. These results are not meant to offer a precise description or capturing of these experiences. Rather, we sought descriptions of how women remembered their early experiences with male perpetrated sexual communications online. It is possible that these were not the very first such experiences, but certainly they did occur at quite young ages and they were remembered vividly. The fairly consistent nature of these stories reduces concerns about these distortions, although there may be some idiopathic variations in recall. Of note, these experiences tended to correspond to periods of adolescence, which is associated with a ‘reminiscence bump’ in adulthood - memories formed during this time period tend to be particularly vivid and accessible (e.g., see Munawar et al., 2018 for a meta-analysis). This has been attributed to brain maturation and cognitive abilities that maximize one's ability to form and retain information (Janssen & Murre, 2008).

Transgender and non-binary participants were excluded from these analyses, as this is a starting point to investigate the experiences of women with male-perpetrated harassment. The nature and timing of one's transition or gender expression was not known and may have affected their experiences with TFSV. It is possible that they would experience unique forms of online harassment that may have differed from those of non-transgender participants or those who identify with binary gender. Another limitation pertains to the sample employed. As we limited this investigation to opposite-sex harassment, potential TFSV from other girls and women was ignored. Given that prior work has found that queer, transgender, and nonbinary youth experience higher rates of sexual harassment in person (Mitchell et al., 2014) and online (Gámez-Guadix et al., 2022; Powell et al., 2020), it is imperative that future research should investigate the question of online sexual harassment with more carefully designed questions that can address the experiences of these populations.

This research focused on the experiences of girls with stranger sexual harassment due to existing gender role norms that normalize and perpetuate female objectification (Baptist & Coburn, 2019; Vera-Gray, 2016). There is work to show that boys and late adolescent men do receive unwanted sexual communications from women as well, also beginning at early ages and causing emotional distress and confusion (Flynn et al., 2024). We acknowledge the very real experience of male victimization and note that unique considerations may apply to those experiences. Further research is warranted on both fronts.

Further, our current assessment focused on cisgender girls and women, but we note that non-binary and transgender persons may be at higher risk of victimization (Mitchell et al., 2014). This, too, requires a careful lens and considerations of harassment that may be particular to gender diverse communities. We would also recommend exploring TFSV at the intersection of gender, race, religion, sexual orientation, or other social identities. Fredrickson and Roberts (1997) noted that the experience of sexual objectification has typically overlooked diversity in female populations, over-relying on White, middle class girls and women. Along these lines, Bailey and Burkell (2021) note that we must consider TFSV within systems of oppression, including multiple social identities that may overlap to create a differential risk of violence and victimization. Finally, these findings are particular to a North American context. Other research has investigated TFSV in other countries, with rates ranging from 2% to 84%, varying by country in a recent meta-analysis with a global average of 30% (Benitez-Hidalgo et al., 2025). It is difficult to make cross-national comparisons due to measurement differences, cultural variations around gender norms, tolerance of sexual communication, and digital access.

## Conclusions

Ultimately, a change in the way we think about online sexual communications between strangers is needed, comparable to the way in which attitudes have shifted about drunk driving and indoor smoking. It is possible that some men misread friendly conversations as “flirting” or tacit approval to send explicit content. The lack of clarity in these interactions emphasizes the need for clear boundaries when communicating in online spaces. It appears that stranger sexual harassment of adolescent girls is a widespread experience that begins early, happens often, and has negative consequences. In many cases reported herein, these older men sought out and knew they were communicating with children and adolescent girls. Children and youth spaces online may be particularly at risk for such behavior, suggesting a need for more effective interventions.
